# Extensible on-chip mode manipulations based on metamaterials

**DOI:** 10.1038/s41377-022-00901-w

**Published:** 2022-07-01

**Authors:** Xuanru Zhang, Tie Jun Cui

**Affiliations:** State Key Laboratory of Millimeter Waves and Institute of Electromagnetic Space, School of Information Science and Engineering, Southeast University, Nanjing, 210096 China

**Keywords:** Silicon photonics, Metamaterials

## Abstract

An extensible framework is proposed for on-chip spatial-mode manipulations based on metamaterial building blocks, which enables the excitation of arbitrarily high-order spatial modes in silicon waveguides. It makes a significant step towards the comprehensive and on-chip manipulations of spatial lights, and may provide promising opportunities for complex photonic functionalities.

Mode-division multiplexing (MDM) technique, which employs different fiber modes as different channels, is recognized as a powerful tool to increase the data capacity of optical fiber communications^1–3^. Mode-selective manipulations also demonstrate great potentials in diverse information processing fields including optical sensing, neuro-inspired photonic computing, and quantum optical devices. However, the traditional mode manipulation techniques, such as phase matching^4^, beam shaping^5^, etc., are fundamentally limited by extendibility. Each mode operator needs to be specifically analyzed and optimized. Meanwhile, arbitrarily high-order mode manipulations are inherently restricted by either the working principle or the available fabrication technologies.

Metamaterials, which are constructed by subwavelength artificial structures and present extraordinary electromagnetic properties, have attracted significant interests for their fabulous wavefront controlling capabilities in subwavelength scales^6^. For on-chip light manipulations^7^, metamaterials enable mode conversions within a short interacting distance and significantly reduce the device footprints^8–10^. Broadband operations can be designed and realized using the metamaterials^11,12^.

Written in this issue of *Light: Science & Applications*, Xuhan Guo and Yikai Su’s research team at Shanghai Jiao Tong University in China proposes an extendible framework for mode manipulations of arbitrary orders, based on programming the topological arrangement of the metamaterial building blocks (BBs)^12,13^. A high-order mode operator up to the 20^th^ has been implemented. As a proof of concept application, a metamaterial BBs-based MDM circuit is accomplished on a silicon-on-insulator (SOI) wafer and is demonstrated for high-speed data transmissions of 8-channel 16-quadrature amplitude modulation (16-QAM) signals with a data rate of 813 Gb/s.

The 3D schematics for the even-order and odd-order mode operators are demonstrated in Fig. 1a and b, respectively. The basic metamaterial BB is marked in the dashed box in Fig. 1a, which is a TE_0_-TE_2_ mode operator exploiting fully-etched dielectric slots on a silicon wafer. The TE_0-_TE_2_ metamaterial BB is of a symmetric arrow-like shape^14^, which leads to strong interactions between involved modes and a compact footprint of 1.23 × 2.7 μm^2^. Mode converters of arbitrary order can be implemented by programming multiple metamaterial BBs in a simple parallel layout. Considering the mode symmetry, even-order mode operators require N/2 metamaterial BBs (N is the mode order), i.e., N dielectric slots. For odd-order mode converter, (N + 1)/2 metamaterial BBs are required with the last unit in an “incomplete BB” geometry. A series of high-order mode converters are fabricated and experimentally characterized by the excess loss (EL) and the crosstalk (CT). In the 1540 nm to 1570 nm wavelength range, the typical EL and CL values are below 3.8 dB and −7 dB for the TE_0_-TE_10_ mode converter.

**Fig. 1:**
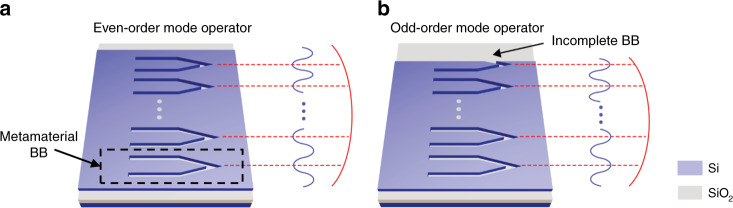
3D schematics for the mode operators. 3D schematics for the even-order (**a**) and odd-order (**b**) mode operators, respectively. The dashed box in (**a**) denotes a metamaterial BB. Reproduced from ref. 12, by permission from Springer Nature, Light: Science & Applications.

The proposed concept of metamaterial BBs breaks the long-standing difficulties in flexibly extensible mode manipulations, and enables the excitation of arbitrarily high order spatial modes in a silicon waveguide. The significant extensibility marks a significant step towards the comprehensive manipulation of light on-chip, and may provide promising opportunities for complex photonic functionalities. The topological arrangement of high-order mode operators allows a user-friendly designing process. In addition, the low EL, low CT, broad bandwidths, compact footprints, and simple fabrication processes endow promising applications for the proposed scheme. In a broad sense, the proposed framework based on metamaterial BBs can be extended to other semiconductors including the indium phosphide or the silicon nitride technologies, and other frequency regimes such as the mid-infrared band.
